# Genome‐wide analysis of long noncoding RNA (lncRNA) expression in colorectal cancer tissues from patients with liver metastasis

**DOI:** 10.1002/cam4.738

**Published:** 2016-05-11

**Authors:** Dong Chen, Qiang Sun, Xiaofei Cheng, Lufei Zhang, Wei Song, Dongkai Zhou, Jianjiang Lin, Weilin Wang

**Affiliations:** ^1^ Department of Colorectal SurgeryFirst Affiliated HospitalSchool of MedicineZhejiang UniversityHangzhouZhejiangChina; ^2^ Division of Hepatobiliary and Pancreatic SurgeryDepartment of SurgeryFirst Affiliated HospitalSchool of MedicineZhejiang UniversityHangzhouZhejiangChina; ^3^ Key Laboratory of Combined Multi‐Organ TransplantationMinistry of Public HealthHangzhouZhejiangChina; ^4^ Key Laboratory of Organ TransplantationHangzhouZhejiangChina

**Keywords:** colorectal cancer (CRC), long noncoding RNA (lncRNA), NAD (P) H Dehydrogenase, Quinone 2 (NQO2)

## Abstract

The liver is the most frequent site of metastasis in colorectal cancer (CRC), in which long noncoding RNAs (lncRNAs) may play a crucial role. In this study, we performed a genome‐wide analysis of lncRNA expression to identify novel targets for the further study of liver metastasis in CRC. Samples obtained from CRC patients were analyzed using Arraystar human 8 × 60K lncRNA/mRNA v3.0 microarrays chips to find differentially expressed lncRNAs and mRNAs. The results were confirmed by quantitative reverse transcription‐polymerase chain reaction (qRT‐PCR). The differentially expressed lncRNAs and mRNAs were identified through fold change filtering. Gene ontology (GO) and pathway analyses were performed using standard enrichment computational methods. In the CRC tissues from patients with liver metastasis, 2636 lncRNAs were differentially expressed, including 1600 up‐regulated and 1036 down‐regulated over two‐fold compared with the CRC tissues without metastasis. Among the 1584 differentially expressed mRNAs, 548 were up‐regulated and 1036 down‐regulated. GO and pathway analysis of the up‐regulated and down‐regulated mRNAs yielded different results. The up‐regulated mRNAs were associated with single‐organism process (biological process), membrane part (cellular component), and transporter activity (molecular function), whereas the down‐regulated mRNAs were associated with cellular process, membrane, and binding, respectively. In the pathway analysis, 27 gene pathways associated with the up‐regulated mRNAs and 51 gene pathways associated with the down‐regulated mRNAs were targeted. The significant changes in NQO2 (NM_000904) mRNA and six associated lncRNAs were selected for validation by qRT‐PCR. Aberrantly expressed lncRNAs may play an important role in the liver metastasis of CRC. The further study can provide useful insights into the biology and, ultimately, the prevention of liver metastasis.

## Introduction

Although the incidence of colorectal cancer (CRC) in both men and women is decreasing 1, CRC remains the third most common cancer and the third leading cause of cancer death in men and women in the United States 2. The survival of patients with CRC is closely related to the occurrence of metastasis, especially to the liver 3, 4. Indeed, hepatic metastases are observed in 75–83% of all metastatic CRC 5, 6, 7. Lack of specificity and sensitivity precludes the application of all existing serum markers, such as carcinoembryonic antigen (CEA), for the early detection of CRC 4, 8. Diagnosis of CRC patients with liver metastases still depend on radiology and biopsy 4. Nevertheless, it is well known that the radiology guided biopsy also has its limitation of sensitivity. Therefore, in order to improve the survival of CRC patients, exploring new biomarkers and the molecular mechanisms underlying metastatic progression in CRC is expected that may lead to the earlier diagnosis and treatment of patients with liver metastases.

Recent studies have revealed that noncoding RNAs (ncRNAs), including ribosomal RNA (rRNA), transfer RNA (tRNA), microRNA (miRNA), and long noncoding RNA (lncRNA), participate in many biological and pathological processes 9, 10. In contrast to small ncRNAs, lncRNAs contain 200–100,000 nucleotides in length. Although the recent application of next‐generation sequencing to a growing number of cancer transcriptomes has indeed revealed thousands of lncRNAs whose aberrant expression is associated with different cancer types, few of which have been functionally characterized 11. lncRNAs have key roles in gene regulation and thus affect various aspects of cellular homeostasis, including proliferation, survival, migration, or genomic stability 11. Moreover, there is increasing evidence that many lncRNAs play significant roles in the regulation of CRC 12.

Among these lncRNAs, the up‐regulation of HOTAIR, MALAT1, CCAT2, and the down‐regulation of LOC285194, UC.388, and LET have been implicated in promoting metastasis of CRC 12. However, the biological and pathological functions of most lncRNAs are still unclear. In this study, we examined differentially expressed lncRNAs and mRNAs in tissues obtained from CRC patients with (experimental group, Exp G) and without (control group, Ctrl G) liver metastasis, aiming to identify novel diagnostic and prognostic markers in CRC patients with liver metastasis.

## Materials and Methods

## Samples

Samples were collected from patients in the Department of Colorectal Surgery, First Affiliated Hospital, Zhejiang University, between October 2014 and May 2015. Twelve primary CRC samples were obtained from six patients with CRC and liver metastasis (Exp G) and six patients with CRC without metastasis (Ctrl G) (Table 1). None of the patients had received neoadjuvant therapy, and the samples were pathologically confirmed postoperatively as colorectal adenocarcinoma. The samples were taken within 10 min after tumor excision, immediately immersed in RNA later^®^ stabilization solution (Thermo Fisher Scientific, Carlsbad, CA, USA), and then stored at −80°C until used in the experiments. Written informed consents were obtained from all patients and this study was approved by the Ethics Committee of the First Affiliated Hospital, College of Medicine, Zhejiang University.

**Table 1:** List of the clinical information

No.	Age (yrs)	Gender	Primary location	Metastasis	Disease duration	Pathology grade^a^	Tumor size (cm)	Clinical stages^b^	Operation method^c^
Exp1	52	F	Descending colon	Liver	1 month	PDA	7.5 × 3.5	T4N2M1	LPH + LLH
Exp2	72	M	Descending colon	Liver	15 days	MA	6.0 × 3.0	T4N1M1	LLH + BLM
Exp3	62	M	Rectum	Liver	4 months	MA	3.5 × 2.5	T3N1M1	LAR + LPH
Exp4	68	M	Rectum	Liver	2 month	PDA	2.5 × 2.0	T3N2M1	AR + PH
Exp5	64	M	Ascending colon	Liver	8 month	PDA	4.0 × 4.01	T4N2M	RH^d^
Exp6	66	F	Sigmoid colon	Liver	10 day	MA	4.0 × 3.5	T4N2M1	R SCC^d^
Ctrl1	81	M	Descending colon	No	4 month	MA	5.0 × 4.0	T3N2M0	LLH
Ctrl2	50	M	Ascending colon	No	5 month	PDA	3.0 × 3.00	T3N1M	RH
Ctrl3	56	M	Ascending colon	No	2 month	MA	8.0 × 4.5	T4N0M0	RH
Ctrl4	64	M	Descending colon	No	2 month	MDA	6.0 × 5.5	T3N0M0	LLH
Ctrl5	84	M	Ascending colon	No	3 month	MA	3.0 × 3.0	T2N0M0	LRH
Ctrl6	78	F	Ascending colon	No	12 month	MA	12.0 × 12.0	T3N0M0	RH

Exp G, Experimental group; Ctrl G, Control group.

a MA, Mucinous adenocarcinoma; MDA, Moderately differentiated adenocarcinoma; PDA, Poorly differentiated adenocarcinoma.

b It includes lymphatic metastasis as N stage.

c AR, Anterior resection; BLM, Biopsy of liver metastases; LAR, Laparoscopic anterior resection; LLH, Laparoscopic left hemicolectomy; LPH, Laparoscopic partial hepatectomy; LRH, Laparoscopic right hemicolectomy; PH, Partial hepatectomy; RH, Right hemicolectomy; RSCC, Resection of sigmoid colon cancer.

d The liver metastasis had been confirmed by preoperative liver biopsy.

## RNA isolation and quality control

Total cellular RNA was isolated from each sample using a homogenizer (IKA, Germany) and TRI zol reagent (Invitrogen, Carlsbad, CA) and then purified using the RNeasy mini kit (Qiagen, Hilden, Germany) according to the manufacturer's protocol. The purified RNA was quantitated using the Nano Drop ND‐1000 spectrophotometer (Thermo Fisher Scientific) and its quality was assessed using the Agilent 2100 Bioanalyzer (Agilent Technologies, Santa Clara, CA, USA).

## Microarray analysis

Human 8 × 60K lncRNA/mRNA v3.0 microarrays (Arra‐ystar, Rockville, MD, USA) designed for the global profiling of 30,586 human lncRNAs and 26,109 protein‐coding transcripts from validated public transcriptomic databases (Refseq, UCSC known genes, Gencode, and others) and landmark publications, were used in our study. Each transcript was confirmed using 1–5 probes to improve statistical confidence. The lncRNA expression data have been deposited in the Gene Expression Omnibus database under the accession number GSE75050.

## RNA labeling and array hybridization

Sample labeling and array hybridization were performed according to the protocol provided with the Agilent One‐Color microarray‐based gene expression analysis kit (Agilent Technology). Briefly, mRNA was purified from total RNA by the removal of rRNA (mRNA‐ONLY^™^ eukaryotic mRNA isolation kit, Epicentre). Each sample was then amplified and transcribed into fluorescently labeled cRNA corresponding to the entire length of the transcripts, without 3′ bias, utilizing a mixture of oligo (dT) and random primers (Arraystar Flash RNA labeling kit; Arraystar). The labeled cRNAs were purified using the RNeasy mini kit (Qiagen) while their concentration and specific activity (pmol Cy3/*μ*gcRNA) were determined using the Nano Drop ND‐1000. The labeled cRNAs (1 *μ*g each) were fragmented by adding 5 *μ*L 10 × blocking agent and 1 *μ*L 25 × fragmentation buffer, followed by heating the mixture at 60°C for 30 min. The labeled cRNA was diluted with 25 *μ*L 2 × GE hybridization buffer. Fifty microliters of hybridization solution were dispensed into the gasket slide, which was then assembled onto the lncRNA expression microarray slide. The assembly was incubated for 17 h at 65°C in an Agilent bridization oven. The hybridized arrays were then washed, fixed, and scanned using the Agilent DNA microarray scanner (part number G2505C).

## Data analysis

Agilent feature extraction software (version 11.0.1.1) was used to analyze the acquired array images. Quantile normalization and subsequent data processing were performed using the Gene Spring GX v12.1 software package (Agilent Technologies). Quantile‐normalized lncRNAs and mRNAs in which at least six of 12 samples were flagged as present or marginal (“All Targets Value”) were chosen for further analysis. *P*‐value/FDR filtering was used to identify the lncRNAs and mRNAs whose expression significantly differed between Exp G and Ctrl G. These differentially expressed lncRNAs and mRNAs were identified through fold change filtering. Hierarchical clustering and combined analyses were performed using in‐house scripts.

## Gene function analysis

The GO project provides a controlled vocabulary to describe the genes and gene products of any organism (http://www.geneontology.org). The ontology covers three domains: biological process, cellular component and molecular function. Fisher's exact test is used to determine whether the overlap between the differential expression list and the GO annotation list is greater than would be expected by chance. Pathway analysis is a functional analysis that maps genes to Kyoto Encyclopedia of Genes and Genomes (KEGG) pathways. The *P*‐value (EASE‐score, Fisher's exact test *P*‐value, or hyper geometric *P*‐value) denotes the significance of the GO term and thus the pathway that correlates with the conditions. The lower the *P*‐value, the more significant the pathway and GO term. A *P*‐value ≤ 0.05 is recommended.

## Target mRNA selection and construction of the coding‐noncoding gene co‐expression network

Several mRNAs with normalized high‐intensity expression and a high fold change were selected and then verified by quantitative reverse transcription‐polymerase chain reaction (qRT‐PCR), performed in triplicate. Significantly expressed mRNAs were superimposed onto the lncRNA‐mRNA correlation network to determine their association with the lncRNAs. Those that significantly differed were used in the construction of an lncRNA‐mRNA regulatory network.

In the network, the pink node represents significantly expressed mRNAs and the blue node represents the related lncRNA; the red solid line shows direct connections and a positive correlation between a given lncRNA and a mRNA; the green line indicates their direct connections and a negative correlation.

## qRT‐PCR for selected lncRNAs

Total cellular RNAs were isolated from primary CRC tissues of the two groups using TRI zol reagent (Invitrogen) and then reverse transcribed using the Prime Script RT reagent kit together with gDNA Eraser (Perfect Real Time; Ta Ka Ra, Dalian, China) in accordance with the manufacturer's instructions. According to the microarray results and the analysis of the lncRNA‐mRNA regulatory network, a significantly down‐regulated mRNA (NM_000904; NQO2, NAD (P) H Dehydrogenase, Quinone 2) and 10 correlative lncRNAs [five up‐regulated (NR_046711, NR_004855, NR_036537, NR_036580 and NR_002795) and five down‐regulated (NR_034129, NR_033878, NR_036484, NR_027054 and NR_027242)] were analyzed by qRT‐PCR (quantitative reverse transcription‐polymerase chain reaction) performed in triplicate using a SYBR Green PCR kit (Ta Ka Ra). Glyceraldehyde3‐phosphate dehydrogenase (GAPDH) mRNA was used as an internal control.

## Statistical analyses

SPSS software (version 16.0, Chicago, IL) was used for the statistical analyses. The data were expressed as the mean ± standard deviation. The microarray data variables were compared between the two groups using Student's *t*‐test. Microarray results were evaluated based on fold changes, and a ≥ 2.0‐fold increase/decrease in lncRNAs or mRNAs was considered statistically significant (*P *< 0.05). Expression of the lncRNAs and mRNAs was compared between the two groups using Student's *t*‐test; *P *< 0.05 was considered to indicate statistical significance.

## Results

### Differentially expressed lncRNAs and mRNAs in the tissues of CRC patients with liver metastasis

**Figure 1:**
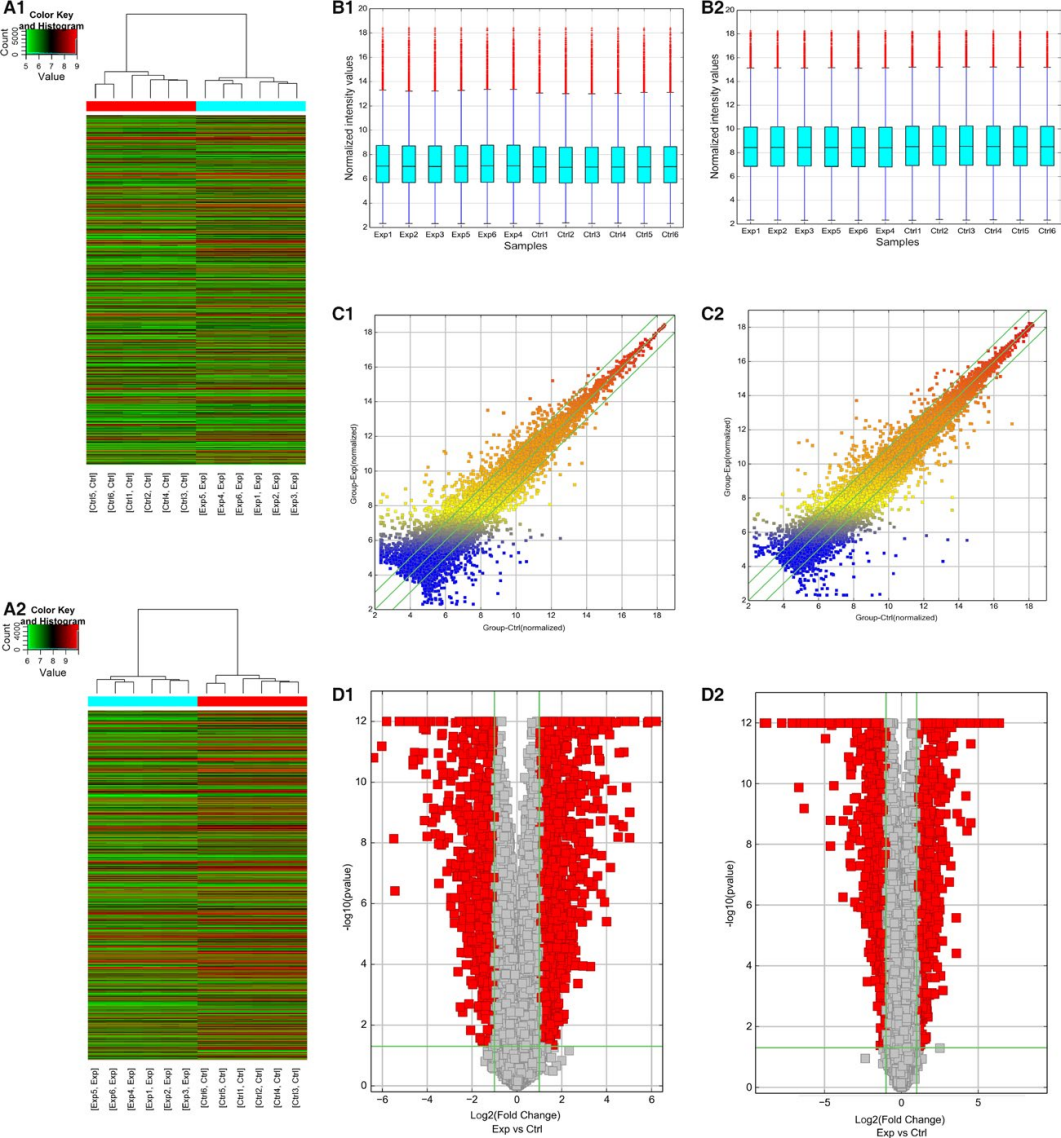
Differentially expressed long noncoding RNAs (lncRNAs) and mRNAs in colorectal cancer (CRC) tissues from patients with Liver Metastasis (Exp G) and without metastasis (Ctrl G). Hierarchical cluster analysis of CRC samples to assess the significant expression of lncRNAs (A1) and mRNAs (A2) based on microarray analyses. Red denotes high relative expression and green low relative expression. Each RNA is represented by a single row of colored boxes and each sample by a single column. The box plot shows the variations in lncRNA (B1) and mRNA (B2) expression. The scatter plot and the volcano plot illustrate the distributions of the data in the lncRNA (C1, D1) and mRNA (C2, D2) profiles. After data normalization, the distributions of the log_2_ ratios among samples were nearly the same. The values of the x‐ and y‐axes in the scatter plot were the averaged normalized signal values of the group (log_2_ scaled). The green lines in the scatter and volcano plots show the significant fold change.

A genome‐wide analysis was performed to profile difference in lncRNA and mRNA expression between Exp G and Ctrl G (Fig. 1). The differences were assessed using authoritative data sources. The 2636 significantly dysregulated lncRNAs (fold change ≥2.0, *P *< 0.05) consisted of 1600 up‐regulated and 1036 down‐regulated lncRNAs (Table S1). In addition, significant differences in the expression of 1584 mRNAs (≥2.0‐fold change, *P *< 0.05) between the two groups were determined. In Exp G, 548 and 1036 mRNAs were significantly up‐ and down‐regulated (≥2.0‐fold change; *P *< 0.05; Table S2).

### Gene ontology and pathway analyses

**Figure 2:**
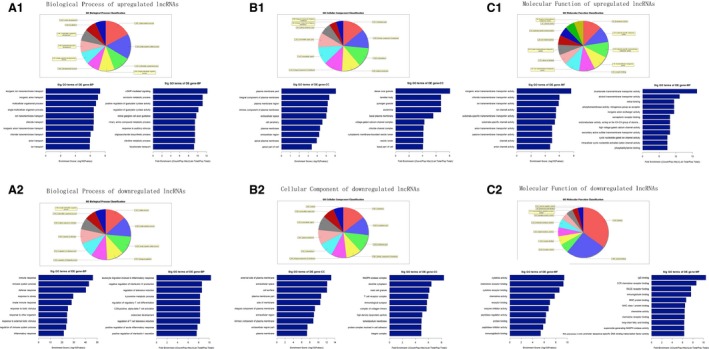
Gene ontology (GO) enrichment analysis of lncRNA‐target genes. GO analysis of lncRNA‐target genes according to biological process (A), cell component (B) and molecular function (C), including analyses of the up‐regulated (A1, B1 and C1) and down‐regulated (A2, B2 and C2) lncRNAs.

**Figure 3:**
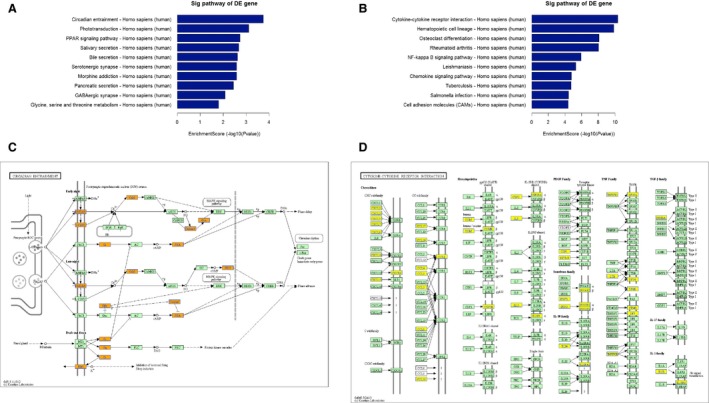
The top 10 enrichment scores in the pathway analysis of the up‐regulated (A) and down‐regulated (B) mRNAs. The lower panel shows the leading pathways associated with the up‐regulated (C) and down‐regulated (D) mRNAs.

In the GO analysis, the up‐ and down‐regulated mRNAs were analyzed separately. In the biological process analysis, single‐organism (341, 17.42%), single‐organism cellular (313, 15.99%) and multicellular organismal (210, 10.73%) were the three most significant processes associated with the up‐regulated mRNAs, while cellular (710, 13.45%), single‐organism (680, 12.88%) and single‐organism cellular (628, 11.9%) were the three most significant processes among the down‐regulated mRNAs. In the cellular component analysis, membrane part (176, 14.08%), intrinsic component of membrane (155, 12.40%) and cell periphery (154, 12.32%) were the three most significant component processes associated with the up‐regulated mRNAs, while membrane (450, 14.65%), cytoplasmic part (369, 12.01%) and membrane part (354, 11.52%) were the three most significant component processes associated the down‐regulated mRNAs. In the molecular function analysis, transporter (52, 12.15%), substrate‐specific transporter (44, 10.28%) and substrate‐specific transmembrane transporter (43, 10.05%) activities were the three most significant functions of the up‐regulated mRNAs, whereas binding (626, 34.95%), protein binding (465, 25.96%) and receptor activity (106, 5.92%) were the three most significant functions of the down‐regulated mRNAs. In the GO analysis, the enrichment score and fold enrichment of these up‐regulated and down‐regulated mRNAs also differed (Fig. 2 and Table S3). Pathway analysis identified 27 and 51 gene pathways targeted by the up‐ and down‐regulated mRNAs, respectively (Fig. 3 and Table S4).

### Target mRNAs selection and coding‐noncoding gene co‐expression network construction

**Figure 4:**
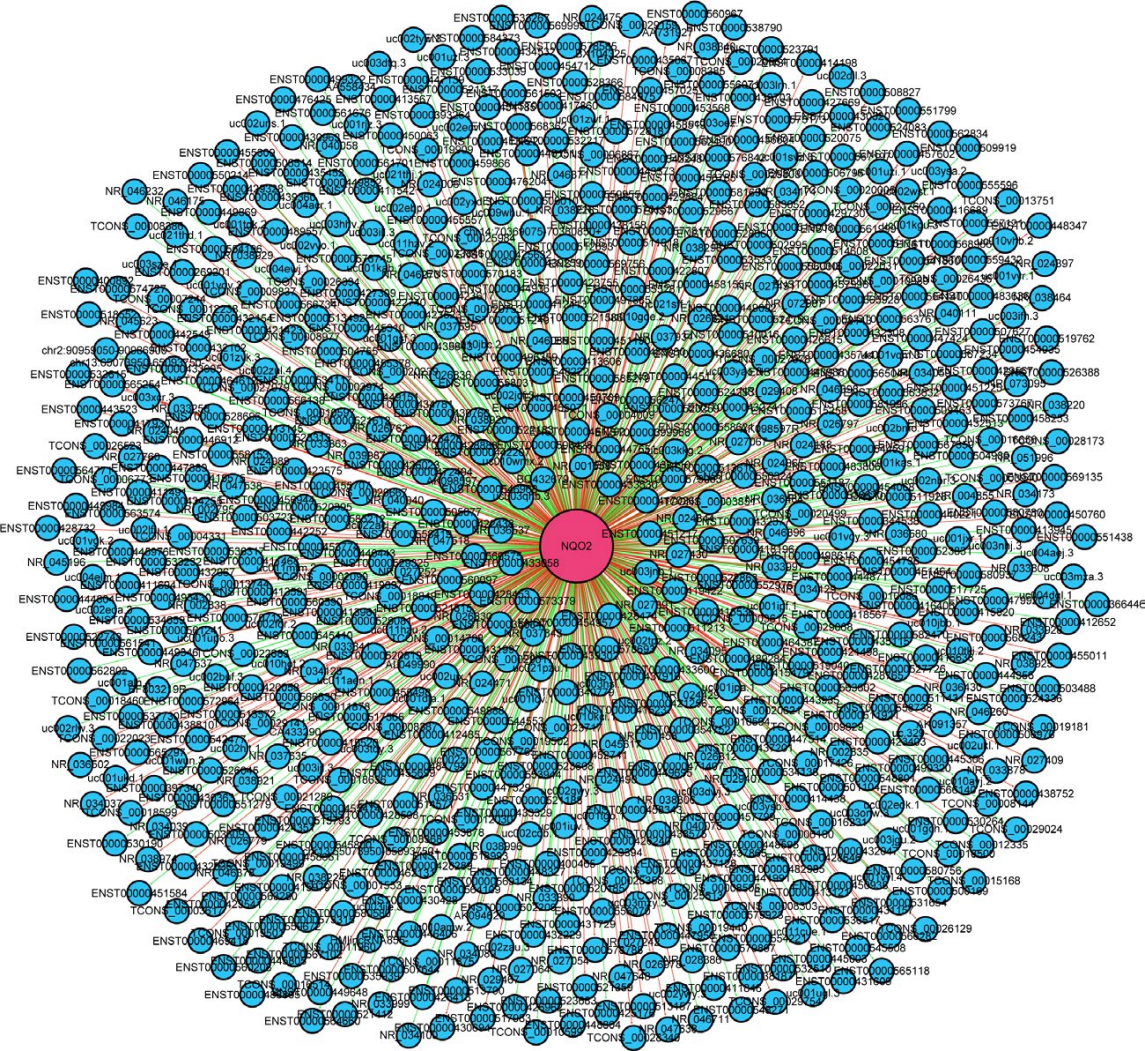
Predictions of the coding‐noncoding gene co‐expression network. The co‐expression network was composed of one mRNA and 769 potentially associated lncRNAs. The 340 direct connections, indicated by the red solid line, are those in which the correlation between the mRNA and the potentially associated lncRNAs was positive. The 429 direct connections, indicated by the green solid line show the respective of negative correlations.

lncRNAs are involved in the regulation of gene expression at the transcriptional, epigenetic, and posttranscriptional levels. Therefore, in this study, the lncRNAs potentially related to liver metastasis in CRC were expected to be associated with the differentially expressed mRNAs identified. Accordingly, from these differentially expressed mRNAs, 10 mRNAs have high normalized intensities and high fold changes (NM_012330, NM_014389, ENST00000347619, ENST00000312942, NM_002247, NM_006103, NM_000904, NM_015032, NM_001008493, and NM_000577) and they were further verified by qRT‐PCR. However, significant expression was confirmed only for NM_000904 (NQO2) (Table 2). Using Pearson's correlation coefficient analysis (*P* ≥ 0.95), we identified 769 potentially associated lncRNAs in the database. We then integrated the predicted potential lncRNA targets with the differentially expressed mRNA‐NQO2 (NM_000904) to construct a coding‐noncoding gene co‐expression network (Fig. 4). Detailed information is presented in Table S5.

**Table 2:** The expression of selected mRNAs in the verification of qRT‐PCR

Selected mRNA	Exp G	Ctrl G	Fold change^a^	*P*‐value
NM_012330	1.35E‐2 ± 6.06E‐3	1.08E‐2 ± 4.89E‐3	1.25	0.416
NM_014389	4.11E‐4 ± 1.50E‐4	6.10E‐4 ± 2.28E‐4	0.68	0.105
NM_002247	3.85E‐4 ± 2.99E‐4	7.59E‐4 ± 4.60E‐4	0.51	0.126
NM_006103	4.20E‐1 ± 7.56E‐1	2.49E‐2 ± 2.40E‐2	16.67	0.259
NM_000577	1.31E‐2 ± 7.52E‐3	2.79E‐2 ± 2.81E‐2	0.47	0.249
NM_000904	3.62E‐3 ± 1.53E‐3	9.42E‐3 ± 5.48E‐3	0.38	0.026^b^
NM_015032	1.84E‐3 ± 9.41E‐4	9.32E‐4 ± 6.92E‐4	1.96	0.080
NM_001008493	1.22E‐2 ± 7.86E‐3	1.46E‐2 ± 6.65E‐3	0.83	0.538
ENST00000312942	3.24E‐3 ± 3.36E‐3	2.15E‐3 ± 2.34E‐3	1.52	0.562
ENST00000347619	1.61E‐3 ± 1.34E‐3	1.61E‐3 ± 7.97E‐4	1.00	0.992

Exp G, Experimental group; Ctrl G, Control group;

a >1 means up‐regulated, <1 means down‐regulated;

b *P* < 0.05, indicates a significant difference between two groups.

### qRT‐PCR validation of the co‐expression network genes

**Figure 5:**
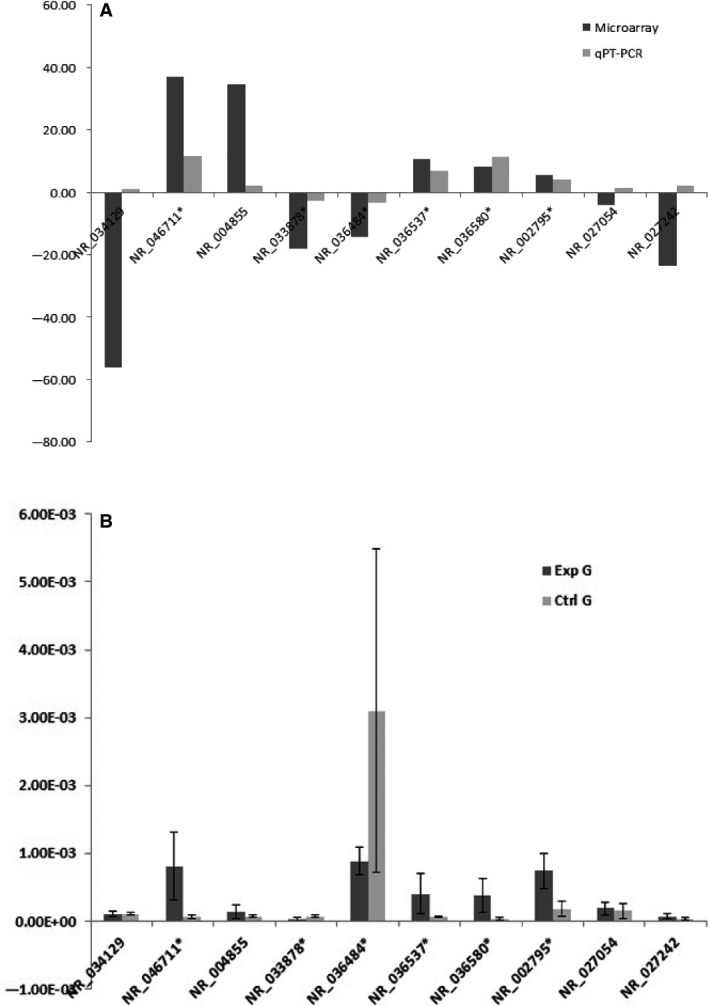
Quantitative reverse transcription‐polymerase chain reaction (qRT‐PCR) validation of 10 randomly selected differentially expressed lncRNAs. Six of the qRT‐PCR‐validated lncRNAs (NR_046711, NR_036537, NR_036580, NR_002795, NR_033878, and NR_036484) showed the same fold change tendencies as those in the microarray results (*P < 0.05). The differences in the other four qRT‐PCR‐validated lncRNAs (NR_034129, NR_004855, NR_027054 and NR_027242) were not statistically significant. (A) Comparison of the microarray data and qPCR results; (B) Comparison of colorectal cancer tissues from patients with Liver Metastasis (Exp G) and without metastasis (Ctrl G).

Five up‐regulated (NR_046711, NR_004855, NR_036537, NR_036580, and NR_002795) and five down‐regulated (NR_034129, NR_033878, NR_036484, NR_027054, and NR_027242) lncRNAs were selected from the 12 CRC samples for further verification by qRT‐PCR. Four significantly up‐regulated (NR_046711, NR_036537, NR_036580, and NR_002795) and two significantly down‐regulated (NR_033878 and NR_036484) lncRNAs showed the same fold change patterns as those in the microarray results. The fold changes of the other four lncRNAs (NR_034129, NR_004855, NR_027054, and NR_027242) did not reach statistical significance (Fig. 5).

## Discussion

Among numerous molecules demonstrated to play roles in CRC, lncRNAs have been the focus of increasing attention for their aberrant expression in carcinogenesis 13. In recent years, several lncRNAs have been found to be associated with metastasis of CRC. A previous study indicates that the role of the HOTAIR in CRC progression is associated with the acquisition of stemness, with the potential mechanism of gene silencing by binding to PRC2 and LSD1 14, 15. The expression of metastasis‐associated lung adenocarcinoma transcript 1 (MALAT1) was found to be associated with CRC metastasis 12, and the down‐regulation of MALAT1 by resveratrol could decrease the nuclear localization of *β*‐catenin and attenuate Wnt/*β*‐catenin signaling, thereby inhibiting CRC invasion and metastasis 16. CRC‐associated transcript 2 (CCAT2) was reported to be highly overexpressed in microsatellite‐stable CRC, and to promote tumor growth and metastasis by regulating Myc and Wnt 17. Except for the above mentioned lncRNAs which promoted the metastasis of CRC, LOC285194, and uc.388 were shown to be a potential inhibitor and the signal pathway should be further characterized 18, 19. Nevertheless, most lncRNAs expected to be prognostic or predictive in cancer patients have failed to perform these functions when tested in vivo 12. As only a few lncRNAs have been well characterized 12, our approach provides a shortcut to identify novel targets among the many as yet uncharacterized lncRNAs. Indeed, the 2636 significantly differentially expressed (f ≥ 2.0‐fold change) lncRNAs in Exp G contain abundant information worthy of further study.

In previous studies, the abnormal expression of lncRNAs determined in CRC tissue samples was interpreted as indicative of their functional role in the underlying biological process 15, 20, 21. Our microarray results similarly showed the differential expression of numerous lncRNAs and mRNAs in Exp G vs. Ctrl G. GO analysis provides a discrete list of terms to describe the characteristics of gene products as well as functional annotation data from GO consortium members. In our GO analysis, the mRNAs were assigned with respect to the biological process, cellular component and molecular function associated with the most highly correlated coding genes 13. Unlike in previous studies 13, 22, the up‐ and down‐regulated mRNAs in this investigation were analyzed separately in both the GO and pathway analyses. This allowed the detection of obvious differences between the up‐and down‐regulated mRNAs. Using this approach, we tentatively identified several novel factors that may play important roles in the liver metastasis of CRC. In previous studies, MAPK signaling and KITLG, both of which participate in the circadian entrainment pathway, identified in association with the up‐regulated mRNAs, and the cytokine–cytokine receptor interactions, determined for down‐regulated mRNAs, were shown to be associated with the development of CRC cells 23, 24. Although lncRNAs exert diverse effects on the regulation of coding gene expression 25, 26, the exact mechanism remain to be identified.

Since the primary function of lncRNAs is the epigenetic regulation of protein‐coding genes 26, 27, detecting the expression of the target coding gene is an effective way to reveal the putative functions of lncRNAs 13. Therefore, as part of our study of lncRNAs, we also analyzed the mRNAs isolated from the two groups. Ten mRNAs had a high normalized intensity and high fold change, but after qRT‐PCR verification, only one (NM_000904, NQO2) was conformed to be significantly expressed.

The human NQO2 gene, located on chromosome 6p25.2, comprises seven exons (the first is noncoding) spanning 19.8 kb. NQO2 encodes a protein of 231 amino acid sand contains 254 single‐nucleotide polymorphisms within 10 kb of the 5′‐end of the first exon and 2 kb of the 3′‐end of the last exon 28. The traditional function of NQO2 was assigned as a phase II detoxification enzyme to protect cells against free radicals and toxic oxygen metabolites 28. The role of NQO2 as a tumor suppressor was raised from the studies demonstrated that NQO2 involves in the control of 20S proteasome‐mediated degradation of p53/p63 and regulates the stability of proteins 29, 30, 31. Furthermore, NQO2 was reported to regulate the stability of cyclin D1 in CWR22Rv1 prostate cancer cells by AKT/GSK‐3*β* signal pathway 32. In addition, NQO2 polymorphism was strongly associated with esophageal cancer 33 and the lymph node metastasis of papillary thyroid microcarcinoma 34. NQO2 was mostly shown to be a negative modifier of carcinogenesis in breast cancer 35, 36, 37, skin neoplasms 38, 39 prostate cancer 32, 40, radiation‐induced B‐cell lymphomas 41 and melanoma 42 while only one study suggested it to be a susceptibility gene for breast carcinogenesis 43. Nevertheless, there have been no direct reports on its altered expression in CRC, both carcinogenesis and metastasis. In this study, NQO2 was down‐regulated significantly in Exp G, suggesting its role in suppressing CRC metastasis. We also identified 769 significantly expressed lncRNAs associated with NQO2, which were integrated into a coding‐noncoding gene co‐expression network. Among the 10 qRT‐PCR‐verified lncRNAs, six showed the same significant tendency as those in the microarray results. However, for three other lncRNAs, the opposite tendency was determined, but the results were not statistically significant and the fold changes were relatively low. Further study of these lncRNAs based on a larger sample size is therefore needed. In a subsequent pathway, analysis aimed at associating the differentially expressed lncRNAs with their target genes, 27 gene pathways were associated with the up‐regulated lncRNAs and 51 with the down‐regulated lncRNAs.

In summary, in this work, we identified a series of differentially expressed lncRNAs in CRC patients with liver metastasis and without any metastasis. Of the potential lncRNAs predicted by bioinformatics analyses to be differentially expressed in the respective tissues, several were verified by qRT‐PCR. These differentially expressed lncRNAs provide novel targets for studies on the pathways and mechanisms of metastatic CRC and thus for the development of therapeutics for its diagnosis and chemoprevention.

## Conflict of Interest

None declared.

## Supporting information


**Table S1**. Differentially expressed lncRNAs in Exp G. (more than two‐fold; *P *< 0.05).

**Table S2**. Differentially expressed mRNAs in Exp G. (more than two‐fold; *P *< 0.05).

**Table S3**. Functional classification of the target genes by GO analysis.

**Table S4.** Pathway Enrichment analysis.

**Table S5.** The associated lncRNAs in CNC‐network.
